# Suitability and effectiveness of marker-assisted selection in soybeans under Asian soybean rust pressure

**DOI:** 10.1007/s11032-026-01694-1

**Published:** 2026-07-07

**Authors:** Claudio Guilherme Portela de Carvalho, Cosme Damião Cruz, Carlos Alberto Arrabal Arias, Aaron Joel Lorenz

**Affiliations:** 1 Embrapa Soja, Caixa Postal 231, Rodovia Carlos João Strass, Distrito de Warta, Londrina, PR CEP 86001-970 Brazil; 2 Laboratório de Bioinformática, Universidade Federal de Viçosa, Av. P H Rolfs, s/n, Campus Universitário, Viçosa, Minas Gerais 36570-000 Brazil; 3 Department of Agronomy and Plant Genetics, University of Minnesota, 411 Borlaug Hall, 1991 Upper Buford Circle, Saint Paul, MN 55108 USA

**Keywords:** *Glycine max* L., Genomic selection, Predictive ability, Quantitative trait loci, Seed yield

## Abstract

Quantitative trait loci (QTL) may explain only a small portion of the phenotypic variation of a trait, limiting their use in breeding programs. This study assessed the effectiveness of marker-assisted selection (MAS) in the F2 population under Asian soybean rust (ASR) pressure for identifying promising F2:5 progenies, even when QTL only explain a small proportion of the phenotypic variation. In addition, we identified SNPs which influence the predictive ability of genomic selection (GS) models to assess whether MAS may be a suitable approach for selecting superior progenies. Evaluated traits included seed yield (SY), days to maturity (DM), days to flowering (DF), plant height (PH), and 50-seed weight (50SW). QTL were identified via simple interval mapping, and genomic predictions were generated using G-BLUP. Models including SNPs associated with QTL on chromosomes 6 and 16 performed as well as or better than genome-wide SNP-based models, indicating that MAS may be a suitable approach. Chromosome 6 was more strongly associated with SY, DM, DF, and PH, while chromosome 16 was associated with 50SW. The CC (EMB-235706-CT on chromosome 6) and AA (EMB-227730-AG on chromosome 16) genotypes in the F2 population showed higher SY and 50SW in F2:5, along with reduced DM, DF, and PH, particularly in the presence of major-effect genomic regions, despite some QTL explaining less than 30% of phenotypic variation. These findings support the development of soybean cultivars with improved agronomic performance for managing ASR.

## Introduction

The accuracy of selecting soybean agronomic traits can be enhanced through QTL mapping (Watanabe et al. 2004; Rodrigues et al. 2010; Li et al. 2017; Santos et al. 2018; Zhu et al. 2021). Although several QTL associated with SY, DM, DF, PH, and 50SW have been identified, some of them only explain a small portion of phenotypic variation. This limitation, combined with stringent significance thresholds used for QTL detection, reduces the effectiveness of MAS, particularly for SY (Kim et al. 2012). In contrast, genomic selection (GS) utilizes genome-wide markers to predict trait performance, capturing effects from all loci regardless of the QTL effect size (VanRaden 2008).

QTL analysis and GS can be combined to strengthen decision-making in breeding programs. For example, excluding SNPs from GS models helps assess their effect on predictive ability. If certain markers are found to be irrelevant, removing them can reduce model dimensionality and computational demands (Silva et al. 2022). In turn, if retaining only markers associated with QTL regions does not compromise model performance, MAS may be a suitable approach for selecting superior progenies.

Genomic selection may enable early selection of F2 plants which generate promising progenies in advanced generations, thereby reducing the number of segregating progenies and, consequently, optimizing mechanical and financial resources. For example, Carvalho et al. (2025) developed models to predict mean agronomic traits in F2:5 soybean progenies under ASR pressure using F2 genotyping. This disease, caused by *P. pachyrhizi*, represents one of the most severe threats to global soybean production, often resulting in near-complete yield losses in affected regions (Zambolim et al. 2022). Developing cultivars which maintain performance under disease pressure, even if they exhibit susceptibility symptoms, represents a sustainable strategy for ASR management, given the pathogen’s limited sensitivity to fungicides and its strong ability to overcome resistance mediated by major-effect genes (Carvalho et al. 2025).

In this study, we used population data from Carvalho et al. (2025) to identify SNPs which influence the predictive ability of their GS models, assessing whether MAS may be a suitable approach for selecting superior progenies. In addition, we evaluated the effectiveness of MAS in F2 for identifying promising F2:5 progenies, particularly in the presence of major genomic regions with strong effects, even when some QTL only explain a moderate or smaller proportion of the phenotypic variation.

## Materials and methods

### Field trials

The F2 and F2:5 generations were developed from a cross between the elite BRQ16-5409 and BR13-9499 lines and tested in field trials in Londrina, PR, Brazil (latitude 23°11’37” S, longitude 51°11’03” W, and altitude 630 m). The parental lines were developed by Embrapa Soja through pedigree selection. BRQ16-5409 was derived from a cross between the cultivars BRS 284 and W-20, while BR13-9499 originated from a cross between the cultivars BRS 284 and BMX Potência. Only BRQ16-5409 was exposed to ASR pressure during development. Both parental lines lack the Rpp1-Rpp7 genes, which provide resistance to *P. pachyrhizi* (Childs et al. 2018).

The F2 generation consisted of 230 plants. Each parent had 50 repetitions. All were sown in November 2019, totaling 330 plants. These were grown in single-plant hill plots (one hill plot = one plant). The arrangement followed a completely randomized design in the field (Lima et al. 2012). The spacing between hill plots within rows was 20 cm. There was 1.5 m between rows. Two rows of the susceptible BRS Conquista cultivar bordered the trial at a distance of 1.5 m distance and around. This resulted in a spacing of 20 cm between plants in a row and 50 cm between rows.

Each F2 plant generated an F2:3 progeny of six plants. Each plant in these F2:3 progenies produced a single F2:4 plant. The resulting F2:4 plants yielded F2:5 seeds in the greenhouse. The experimental design in November 2022 consisted of segregating progenies evaluated alongside intercalary lines under a completely randomized design. We sowed 230 F2:5 progenies, each with six lines, totaling 1,380 F2:5 lines, and included two parents along with ASR-resistant and susceptible checks (BRS 531 and BRS 523). Each parental line and control cultivar was replicated 30 times. The experiment utilized 1,500 single-row plots, each 3 m long and spaced 0.5 m apart. We added one line of each parent and each check for every 50 F2:5 lines sown. BRS Conquista was sown around the perimeter as a border. Crop management followed recommended soybean requirements (Seixas et al. 2020).

The traits evaluated were seed yield per plant or line (SY), days to maturity (DM), days to flowering (DF), plant height (PH), 50-seed weight (50SW), ASR severity (ASRS), and area under the disease progress curve (AUDPC). The agronomic traits, ASRS, and AUDPC were recorded in the F2 and F2:5 generations.

SY was measured in grams; DF was assessed at the R1 stage as the number of days until 50% of the plants within a row reached flowering; DM was evaluated at the R8 stage, when 95% of the pods were brown and suitable for harvest (seed moisture below 15%); PH was measured at the R8 stage from the soil surface to the tip of the main stem; and 50SW was recorded in grams.

The procedures for maintaining, collecting, preparing, concentrating, and applying the ASR inoculum in F2 plants and F2:5 lines followed Lima et al. (2012). The pathogen was collected from naturally infected field plants during the previous cropping season. This reflected the high pathogenic variability of the fungal population in Londrina, PR, Brazil, which comprises multiple genetically and/or phenotypically distinct populations. The spore suspension was applied only to the borders sown with the susceptible cultivar BRS MGBR-46(Conquista) at the V3 developmental stage. In 2019, the field environmental conditions from 5 days before to 5 days after inoculation were an average relative humidity of 86.4%, an accumulated rainfall of 26.4 mm, and an accumulated global solar radiation of 16 MJ m^− 2^. In 2022, the values were 87.6%, 139.4 mm, and 14.7 MJ m^− 2^, respectively.

ASRS was defined as the percentage of leaf area infected by the pathogen using the graphical scale developed by Franceschi et al. (2020). The scale featured 10 distinct severity levels: 0.2%, 1%, 3%, 5%, 10%, 25%, 40%), 55%, 70%, and 84%. Four assessments were performed in the middle third of the plants at approximately seven-day intervals, with the first assessment made 80 days after emergence when the crop canopy had closed (Isard et al. 2006). The AUDPC for each plot (plant or row) was calculated using the trapezoidal integration method (Madden et al. 2007). The type of leaf lesion was also evaluated and classified as reddish-brown (RB, the resistance lesion) or tan (TAN, the susceptibility lesion) (Zambolim et al. 2022). These assessments enabled quantifying disease and identifying resistant genotypes associated with the unexpected presence of major genes.

The plants were harvested at the R8 stage, dried in a shaded environment, threshed, and weighed individually in 2019 and as a row in 2022.

### Heritability and correlation

The correlations between SY and other agronomic traits were evaluated in F2, F2:5, and between generations, and their significance was verified using the t-test. The narrow-sense heritabilities of these traits at the individual level in the F2 generation were estimated by using the weighted least squares method to determine the genetic components of variance (Mather and Jinks 1984). The variance among plants of each parental cultivar was used to estimate the environmental variance.

### Genotyping

Leaf tissue samples were collected from 230 three-week-old F2 plant seedlings. After being lyophilized and ground, DNA from these samples was extracted using a modified CTAB protocol (Rogers and Bendich 1994). Genotyping was performed by selecting a set of 4,224 SNPs from the previously developed soybean BARCSoySNP6K chip (Song et al. 2020) to fill a sector of the EMBRAPA 65 K Infinium multispecies chip (Grattapaglia et al. in preparation). This chip contains 66,413 SNPs shared among 27 plant and animal species, significantly reducing the cost of genotyping individual samples and enabling generation of high-quality interlaboratory portable SNP data. Genotyping was executed at Neogen/Geneseek (Lincoln, NE). Manifest files and intensity data (.idat files) were obtained from Neogen. SNP genotypes were processed following standard genotyping and quality control procedures (Illumina 2010) and exported in AB format, in which alleles A and T at the SNPs are coded as A, and alleles G and C at the SNPs are coded as B. Marker data for F2 plants were subsequently recoded for linkage mapping and genomic selection analyses: genotypes homozygous for the major allele were coded as 2, heterozygous genotypes as 1, and genotypes homozygous for the minor allele as 0. Markers with a call rate (CR) below 90%, minor allele frequency (MAF) below 5%, or genotype frequencies that deviated significantly from the expected 1:2:1 segregation ratio (based on a Bonferroni-adjusted threshold, α = 0.20) were excluded from the analysis.

### Linkage map and QTL

We constructed two genetic linkage maps using SNP markers. The first map was based on the genotypic and phenotypic data of 230 F₂ plants, following the approach described by Ferreira et al. (2006) and Li et al. (2010). The second map used genotypic data from the F2 population and phenotypic data from the corresponding F2:5 progenies, represented as progeny mean values. The linkage groups (LG) limits were between a minimum LOD score of 3.0 and a maximum recombination percentage of 30%. QTL mapping was performed using simple interval mapping and composite interval mapping methods (Lander and Botstein 1989; Zheng 1994). For the latter, cofactor selection was performed through stepwise regression with entry and exit significance levels of 5% and 10%, respectively (Rodrigues et al. 2010). The QTL positions were defined as the peaks of the maximum LOD score. The additive (a) and dominance (d) effects, the d/a ratio, and the QTL coefficient of determination were estimated according to Cruz (2016).

### Predictive ability

The genomic estimated breeding values (GEBV) for agronomic traits in plants from the F2 and F2:5 generations were calculated using the additive G-BLUP model (VanRaden 2008). The modeling included the genotyping and phenotyping of individual F2 plants, as well as the genotyping of F2 and the phenotyping of F2:5 progeny lines.

The F2 population consisted of 230 plants resulting from a cross between the BRQ16-5409 and BR13-9499 lines. We randomly divided this population into five folds, each containing 46 plants, to estimate GEBV. Four folds, totaling 184 plants, were combined to form a training population. The remaining fold, comprising 46 plants, formed a validation population. We repeated this procedure four more times to create five training and validation populations from the initial division.

The models were obtained using the genotypic and phenotypic values of the agronomic traits of the plants in each training population. These models were used to estimate the GEBV of the plants in the validation populations. The predictive ability of the models was configured as the correlation between the GEBV and the phenotypic values of the plants in the validation population (VanRaden 2008). The mean predictive ability of the models from the five sets of populations was defined as the predictive ability of the initial division.

As with the initial split, we repeated the division of the F2 population into five folds nine more times, resulting in a total of 10 divisions. The mean predictive ability was calculated for each, and then the overall mean and standard deviation for the ten divisions were determined. The same process was done using F2 genotyping and mean F2:5 progeny line values.

### Chromosomal region

SNPs from different chromosomal regions were used to identify those regions that influence the predictive abilities of genomic selection models for agronomic traits assessed in F2 and F2:5. The predictive abilities of the models were calculated using SNPs from all chromosomes, SNPs from all chromosomes excluding those associated with QTL, SNPs from chromosomes associated with QTL, SNPs flanking the QTL, and the addition and removal of SNPs from each chromosome. Tukey’s test compared the predictive abilities of models (*p* < 0.05) (Cruz 2016).

### Marker-assisted selection

F2 plants were ranked based on the phenotypic values of the agronomic traits and classified as A if they were among the 25% shortest, with the shortest cycle, the lowest yield, or the lowest weight of 50 seeds, and were classified as D if they were among the 25% tallest, with the longest cycle, the highest yield, or the highest weight of 50 seeds. F2 plants were classified as B when they were included in the 25% to 50% of plants with the lowest phenotypic values; and classified as C when they were included in the 25% to 50% of plants with the highest phenotypic values. This classification was also established for the mean phenotypic values of F2:5 progenies. Based on this classification, the percentages of A, B, C, and D plants that presented specific SNP genotyping associated with QTL were obtained. These percentages enabled verifying MAS’s effectiveness in selecting the best F2 plants and F2:5 progenies.

### Computational analyses

The GENES software (Cruz 2016) was used to estimate the correlations between agronomic traits and the narrow-sense heritabilities of these traits at the individual level in F2, map and detect QTL, construct additive G-BLUP models, and calculate their predictive abilities.

## Results

### Asian soybean rust

All plants of the parental lines BRQ16-5409 and BR13-9499, the F2 and F2:5 segregating generations from their cross, and the check BRS 523 evaluated in F2:5 showed TAN lesions, typical of plants susceptible to ASR, while the check BRS 531 evaluated in F2:5 showed RB lesions, typical of plants resistant to ASR.

The AUDPC for the F2 plants ranged from 1,015 to 1,662, with ASRS in the fourth evaluation ranging from 70% to 100%. The AUDPC for the BRQ16-5409 and BR13-9499 lines ranged from 1,010 to 1,452 and from 1,120 to 1,610, respectively. The ASRS values in the fourth evaluation ranged from 65% to 90% for BRQ16-5409 and from 85% to 96% for BR13-9499.

The AUDPC for the F2:5 progenies ranged from 600 to 952, with ASRS in the fourth evaluation ranging from 51% to 90%. The AUDPC for the BRQ16-5409, BR13-9499, BRS 523, and BRS 531 lines ranged from 654 to 948, 752 to 1,155, 672 to 1,015, and 140 to 395, respectively. The ASRS values in the fourth evaluation ranged from 70% to 85%, 70% to 90%, 75% to 90%, and 8% to 25%, respectively.

### Correlation and heritability

The correlations between SY and DM, DF, PH, and 50SW in F2 were 0.34, 0.30, 0.44, and 0.03, respectively, and in F2:5 they were − 0.77, −0.72, −0.57, and 0.70, respectively. The correlation for SY between F2 and F2:5 was − 0.24. Narrow-sense heritability estimates at the individual level in F2 for SY, DM, PH, and 50SW were 0.59, 0.79, 0.84, and 0.53, respectively.

### QTL mapping

From a set of 4,224 SNPs derived from the previously developed soybean BARCSoySNP6K chip (Song et al. 2020), 2,071 markers exhibited a MAF greater than 5% and CR above 90%. Of these, 1,904 markers segregated in a 1:2:1 ratio (with Bonferroni correction ((α = 20%), and 1,763 SNPs were non-redundantly mapped. A genetic linkage map was constructed using these 1,763 SNPs, resulting in a total linkage group length of 1,953.02 cM. More than one linkage group was formed for chromosomes 1, 6, 7, 8, 11, and 19.

Mapping using phenotypic and genotypic information from F2 identified QTL in the region between the SNPs EMB-232,017-CA (17,230,927 bp and 30.672 cM) and EMB-235,706-CT (43,235,059 bp and 42.36 cM) (Region 1) and between the SNPs EMB-235,706-CT and EMB-236,672-AC (48,098,064 bp and 71.139 cM) (Region 2) on chromosome 6, and between the SNPs EMB-227,730-AG (1,333,772 bp and 7.231 cM) and EMB-227,756-TC (1,402,063 bp and 7.695 cM) on chromosome 16, associated with DM, DF, PH, and 50SW evaluated in a soybean population derived from the cross between the BRQ16-5409 and BR13-9499 lines, under Asian soybean rust pressure (Table 1). A third QTL was found on chromosome 6, in the region between the SNPs EMB-236,840-TC (48,992,462 bp and 77.123 cM) and EMB-236,861-AC (49,111,467 bp and 77.674 cM) (Region 3), only associated with the first three traits. In addition to the QTL on chromosome 6, a QTL for SY was found on chromosome 13, between the SNPs EMB-233,968-GT (35,950,224 bp and 99.035 cM) and EMB-234,514-CT (38,045,246 bp and 109.115 cM).

**Table 1:** Quantitative trait loci (chromosome (Chr), coefficient of determination (R^2^) and effect) associated with agronomic traits evaluated in a soybean population in F2 and F2:5 under high Asian soybean rust pressure and genotyping in F2

	F2					F2:5				
			QTL effect					QTL effect		
Trait^1^	Chr^2^	R^2^(%)	a^3^	d	d/a	Chr^2^	R^2^(%)	a^3^	d	d/a
SY	6^1^	17.5	−8.8	6.6	−0.8	6^1^	29.3	74.4	1.56	−0.0
	6^2^	18.9	−8.1	7.1	−0.9	6^2^	28.3	65.9	−3.5	−0.1
	6^3^	-	-	-	-	6^3^	7.0	−31.5	−2.3	0.1
	13	5.7	1.3	6.9	5.5	13	-	-	-	-
	16	-	-	-	-	16	15.4	−44.0	−0.1	0.0
DM	6^1^	42.2	−8.9	2.5	−0.3	6^1^	46.6	−7.0	1.1	−0.2
	6^2^	40.3	−8.8	2.7	−0.3	6^2^	45.6	−6.5	1.3	−0.2
	6^3^	7.5	3.6	1.2	0.3	6^3^	9.1	2.9	0.0	0.0
	16	27.2	6.7	0.0	0.0	16	22.3	4.2	−0.1	0.0
DF	6^1^	43.0	−5.5	1.5	−0.3	6^1^	61.0	−9.3	0.6	−0.1
	6^2^	39.7	−6.2	1.9	−0.3	6^2^	58.6	−9.0	1.2	−0.1
	6^3^	5.9	2.1	0.9	0.4	6^3^	10.5	3.7	−0.2	−0.1
	16	19.5	3.9	−0.2	0.0	16	9.2	3.2	−0.5	−0.2
PH	6^1^	47.7	−26.5	5.0	−0.2	6^1^	41.2	−13.1	0.6	0.0
	6^2^	46.9	−25.5	6.8	−0.3	6^2^	41.6	−11.4	1.3	−0.1
	6^3^	9.0	10.8	1.6	0.1	6^3^	9.3	5.4	−0.5	−0.1
	16	18.3	14.7	−1.5	−0.1	16	11.3	5.6	−0.4	−0.1
50SW	6^1^	10.0	0.3	−0.1	−0.4	6^1^	17.2	0.5	0.1	0.1
	6^2^	8.6	0.3	−0.1	−0.4	6^2^	18.1	0.4	−0.1	−0.3
	6^3^	-	-	-	-	6^3^	6.2	−0.5	0.0	0.0
	16	17.3	−0.4	0.1	−0.3	16	31.2	−0.2	0.1	−0.4

^1^SY: seed yield per plant (F2) or line (F2:5) in g, DM: days to maturity, DF: days to flowering, PH: plant height in cm, and 50SW: 50-seed weight in g. ^2^6^1^: region between the SNPs EMB-232,017-CA and EMB-235,706-CT on chromosome 6, 6^2^: region between the SNPs EMB-235,706-CT and EMB-236,672-AC on chromosome 6, 6^3^: region between the SNPs EMB-236,840-TC and EMB-236,861-AC on chromosome 6, 13: region between the SNPs EMB-233,968-GT and EMB-234,514-CT on chromosome 13, and 16: region between the SNPs EMB-227,730-AG and EMB-227,756-TC on chromosome 16. ^3^a: additive effect and d: dominance effect

The determination coefficients (R²) of the QTL associated with Regions 1 (42.2% to 47.7%) and 2 (39.7% to 46.9%) on chromosome 6 were similar for DM, DF, and PH. These values were higher than the R²s (18.3% to 27.2%) of the QTL identified on chromosome 16 and the R²s (below 10%) of the QTL associated with Region 3 on chromosome 6. The R² values of the QTL related to Regions 1 (17.5%) and 2 (18.9%) on chromosome 6 for SY were also higher than the R² value (below 6%) obtained for the QTL on chromosome 13. The relevance of the QTL in Regions 1 (R² = 10.0%) and 2 (R² = 8.6%) on chromosome 6 was less significant for 50SW, with the R² of the QTL on chromosome 16 exceeding 17%.

Mapping using phenotypic information from the means of F2:5 progenies and genotypic information from F2 plants detected the same QTL previously observed using F2 phenotypic data for DM, DF, and PH (Table 1). Unlike F2, these QTL were also found for SY and 50SW when using F2:5 phenotypes. No QTL was found on chromosome 13 for SY in F2:5. The R² of the QTL obtained in F2:5 for DM, DF, PH, and SY showed greater importance for the QTL associated with Regions 1 (29.3% to 61.0%) and 2 (28.3% to 58.6%) on chromosome 6 compared to the QTL associated with chromosome 16 (9.2% to 22.3%) and the QTL in Region 3 on chromosome 6 (7% to 10.5%). Similar to F2, the superiority of the R² in Regions 1 and 2 on chromosome 6 was not observed for 50SW, where the R² of the QTL associated with chromosome 16 (31.2%) was higher than those obtained in Regions 1 (17.2%), 2 (18.2%), and 3 (6.2%) on chromosome 6.

Except for SY in F2, the d/a ratio was close to zero for the agronomic traits evaluated in F2 and F2:5. In turn, the d/a ratio for SY in F2 was close to one for the QTL in Regions 1 and 2 on chromosome 6 and above 1 for the QTL on chromosome 13.

### Predictive ability in F2

The predictive abilities of the models in F2, using additive GBlup and the 1,763 SNPs associated with all chromosomes, were 0.30 for SY, 0.72 for DM, 0.65 for DF, 0.66 for PH, and 0.39 for the 50SW (Table 2). The application of models utilizing the 114 SNPs from chromosome 6 yielded similar predictive abilities to those observed in models employing the full complement of chromosomes (*p* < 0.05). Except for SY, using only 85 SNPs from chromosome 16 resulted in models with predictive abilities greater than 0.35. The predictive ability (0.38) for 50SW using SNPs from chromosome 16 was higher than that with SNPs from chromosome 6 (0.29). Including only these 199 (114 + 85) SNPs in the analysis generated models with greater predictive abilities than those obtained for models including the 1,763 SNPs across all evaluated traits. In contrast, including SNPs from a single chromosome, except for chromosomes 6 and 16, resulted in models with predictive abilities close to zero. Then, when chromosomes 6 and 16 were excluded from the analyses, the predictive abilities of the models were also close to zero.

**Table 2:** Predictive ability of soybean agronomic traits evaluated in a population in F2 and F2:5 under Asian soybean rust pressure

Chromosome region^1^	F2					F2:5				
	SY^2,3^	DM	DF	PH	50SW	SY	DM	DF	PH	50SW
All chromosomes (Chr)	0.30_d_	0.72_b_	0.65_d_	0.66_d_	0.39_de_	0.56_c_	0.72_b_	0.66_d_	0.60_d_	0.64_b_
All chromosomes (Chr) - Chr 6 - Chr 16	0.04_f_	−0.03_i_	0.01_j_	−0.02_j_	0.11_g_	−0.03_j_	−0.02_j_	−0.05_h_	0.14_h_	−0.04_h_
Chr 6	0.38_c_	0.61_d_	0.64_d_	0.62_e_	0.29_f_	0.51_e_	0.66_d_	0.75_b_	0.62_cd_	0.36_f_
Chr 16	0.03_f_	0.49_g_	0.41_h_	0.37_h_	0.38_e_	0.35_h_	0.42_h_	0.24_g_	0.28_g_	0.54_d_
Chr 6 + Chr16	0.36_c_	0.83_a_	0.79_b_	0.80_b_	0.47_b_	0.64_b_	0.82_a_	0.82_a_	0.71_b_	0.66_b_
QTL 1 (Chr 6)	0.42_ab_	0.64_c_	0.67_c_	0.68_c_	0.30_f_	0.53_d_	0.68_c_	0.77_b_	0.64_c_	0.40_e_
QTL 2 (Chr 6)	0.39_bc_	0.59_e_	0.62_e_	0.63_e_	0.28_f_	0.53_de_	0.63_e_	0.72_c_	0.59_d_	0.39_e_
QTL 3 (Chr 6)	0.19_e_	0.27_h_	0.23_i_	0.30_i_	0.13_g_	0.26_i_	0.29_i_	0.32_f_	0.30_g_	0.23_g_
QTL 1, 2 and 3 (Chr 6)	0.42_ab_	0.64_c_	0.65_cd_	0.68_c_	0.28_f_	0.54_cd_	0.67_cd_	0.77_b_	0.64_c_	0.41_e_
QTL 1 (Chr 16)	0.02_f_	0.52_f_	0.45_g_	0.42_g_	0.42_cd_	0.39_g_	0.46_g_	0.30_f_	0.34_f_	0.55_d_
QTL 3 (Chr 6) and QTL 1 (Chr 16)	0.16_e_	0.58_e_	0.50_f_	0.51_f_	0.43_c_	0.45_f_	0.55_f_	0.43_e_	0.44_e_	0.60_c_
SNP (QTL 1 – Chr 6) and SNP (QTL 1 – Chr 16)	0.42_ab_	0.83_a_	0.79_b_	0.81_b_	0.51_ab_	0.66_ab_	0.83_a_	0.83_a_	0.73_ab_	0.69_a_
QTL 1, 2 and 3 (Chr 6) and QTL 1 (Chr 16)	0.42_a_	0.84_a_	0.81_a_	0.82_a_	0.51_a_	0.68_a_	0.84_a_	0.84_a_	0.73_a_	0.70_a_

^1^QTL 1 (Chr 6): between the SNPs EMB-232,017-CA and EMB-235,706-CT on chromosome 6, QTL 2 (Chr 6): between the SNPs EMB-235,706-CT and EMB-236,672-AC on chromosome 6, QTL 3 (Chr 6): between the SNPs EMB-236,840-TC and EMB-236,861-AC on chromosome 6, QTL 1 (Chr 16): between the SNPs EMB-227,730-AG and EMB-228,237-GA on chromosome 16, SNP (QTL 1 – Chr 6): SNP EMB-235,706-CT on chromosome 6, and SNP (QTL 1 – Chr 16): SNP EMB-227,730-AG on chromosome 16. ^2^SY: seed yield per plant, DM: days to maturity, DF: days to flowering, PH: plant height, and 50SW: 50-seed weight. ^2^ Source: Carvalho et al. (2025). ^3^ Means followed by the same lowercase superscript letter do not differ by Tukey’s test at 5% probability

Table 2 also presents the predictive abilities of the models using only the SNPs associated with the QTL for the agronomic traits described in Table 1. The model with the highest predictive ability for SY was generated by including the SNPs EMB-232,017-CA and EMB-235,706-CT, which are associated with the QTL in Region 1. The highest predictive abilities for the other traits were obtained using the SNPs EMB-235,706-C from chromosome 6 and EMB-227,730-AG from chromosome 16. Models including only the SNPs EMB-236,840-TC and EMB-236,861-AC from region 3 of chromosome 6 exhibited non-zero predictive ability. Except for the 50SW, including these SNPs increased the predictive ability of models which had only included the SNPs from chromosome 16, but did not enhance those that had included SNPs from regions 1 or 2 of chromosome 6.

Table 2 also presents the predictive abilities of the models using only the SNPs associated with the QTL for the agronomic traits described in Table 1. The model with the highest predictive ability for SY was generated by including the SNPs EMB-232,017-CA and EMB-235,706-CT, which are associated with the QTL in Region 1. The highest predictive abilities for the other traits were obtained using the SNPs EMB-235,706-C from chromosome 6 and EMB-227,730-AG from chromosome 16. Models including only the SNPs EMB-236,840-TC and EMB-236,861-AC from region 3 of chromosome 6 exhibited non-zero predictive ability. Except for the 50SW, including these SNPs increased the predictive ability of models which had only included the SNPs from chromosome 16, but did not enhance those that had included SNPs from regions 1 or 2 of chromosome 6.

### Predictive ability in F2:5

The models in F2:5 showed varying predictive abilities using different sets of SNPs (Table 2). The predictive abilities for SY, DM, DF, PH, and 50SW when using all chromosomes were 0.56, 0.72, 0.66, 0.60, and 0.64, respectively (Table 2). Using only the 114 SNPs from chromosome 6 resulted in models with similar predictive abilities to those using all chromosomes (*p* < 0.05), and models with 85 SNPs from chromosome 16 generated predictive abilities greater than 0.24. In turn, the predictive ability (0.54) for 50SW using SNPs from chromosome 16 was higher than that with SNPs from chromosome 6 (0.36). Including 199 (114 + 85) SNPs from chromosomes 6 and 16 produced models with similar or greater predictive abilities than those obtained for models including the 1,763 SNPs across all evaluated traits. Excluding chromosomes 6 and 16 resulted in models with near-zero predictive ability.

When only SNPs associated with QTL for agronomic traits were included, the models showed similar or higher predictive abilities than those obtained using all the SNPs from the chromosomes containing those QTL. Using the SNPs EMB-235,706-CT from chromosome 6 and EMB-227,730-AG from chromosome 16 resulted in the highest predictive ability for all traits. Models including only the SNPs EMB-236,840-TC and EMB-236,861-AC from region 3 of chromosome 6 exhibited non-zero predictive ability. Adding these SNPs to the model increased the predictive ability of models which had only included the SNPs from chromosome 16. Still, it did not enhance those that had included only SNPs from regions 1 or 2 of chromosome 6.

### Classification in F2

F2 plants were ranked based on the phenotypic values of agronomic traits and classified as A, B, C, and D, with D representing those with the highest values (Table 3). Plants with the CC genotype at the SNP EMB-235,706-CT on chromosome 6 tended to be classified as short intermediate (B) to short cycle (A), and plants with the TT genotype as long intermediate (C) to long cycle (D). Plants with the same genotype at the SNP EMB-235,706-CT and with the AA genotype at the SNP EMB-227,730-AG on chromosome 16 tend to have a shorter cycle than plants with the GG genotype. For maturation, 79% of plants with the CCAA genotype were classified as short-cycle and 21% as short-intermediate, while 100% of plants with the TTGG genotype were classified as long-cycle. None of the 57 shortest-cycle plants (A) had the TTGG genotype, while none of the 57 longest-cycle plants (D) had the CCAA genotype. Similar results were also observed for DF and PH. Thus, plants with the CC genotype also tended to be classified as intermediate (B) to early flowering and low height, while plants with the TT genotype were classified as intermediate (B) to late flowering and high height. Plants with the same genotype at the SNP EMB-235,706-CT and with the AA genotype tended to have earlier flowering and lower height than plants with the GG genotype.

**Table 3:** Percentage of plants classified based on racking of an F2 population under Asian rust pressure for soybean agronomic traits when considering a specific genotyping of SNPs associated with QTL

SNP1^1^	SNP2	Classification^3^	SY^4^	DM	DF	PH	50SW
CC^2^	AA	A	64	79	86	86	7
		B	29	21	7	14	14
		C	7	0	7	0	7
		D	0	0	0	0	72
CC	AG	A	62	69	69	65	9
		B	29	20	22	26	11
		C	3	11	9	3	34
		D	6	0	0	6	46
CC	GG	A	25	29	37	38	10
		B	55	61	53	52	38
		C	20	10	10	10	33
		D	0	0	0	0	19
CT	AA	A	32	52	35	35	9
		B	10	44	65	52	17
		C	29	4	0	13	30
		D	29	0	0	0	44
CT	AG	A	10	4	6	8	19
		B	17	29	27	34	27
		C	33	54	59	50	31
		D	40	13	8	8	23
CT	GG	A	11	0	3	0	59
		B	35	5	5	8	30
		C	24	38	30	35	8
		D	30	57	62	57	3
TT	AA	A	7	13	6	13	7
		B	7	54	47	33	20
		C	46	33	47	24	40
		D	40	0	0	0	33
TT	AG	A	5	0	0	0	29
		B	14	5	5	0	47
		C	33	29	28	33	24
		D	48	66	67	67	0
TT	GG	A	19	0	0	0	69
		B	24	0	0	0	19
		C	38	0	0	0	12
		D	19	100	100	100	0

^1^SNP1: SNP EMB-235,706-CT on chromosome 6 and SNP 2: SNP EMB-227,730-AG on chromosome 16. ^2^Nitrogenous bases: Thymine (T), Cytosine (C), Adenine (A) e Guanine (G). ^3^A: 25% of F2 plants with shorter height, shorter cycle, lower yield and less 50-seed weight, B: low intermediate, C: high intermediate and D: 25% of F2 plants with greater heights, longer cycle, higher yield and greater 50-seed weight. ^4^ SY: seed yield per plant, PH: plant height, DM: days to maturity, DF: days to flowering, and 50SW: 50-seed weight

The relationships between phenotypes and genotypes were different for 50SW and SY. For 50SW, plants with the AA genotype at the SNP EMB-227,730-AG tended to be classified as great intermediate (C) to great weight of 50 seeds (D), and plants with the GG genotype as low intermediate (B) to low weight of 50 seeds (A). Plants with the same genotype at the SNP EMB-227,730-AG and with the CC genotype at the SNP EMB-235,706-CT tended to have a greater 50SW than plants with the TT genotype. For this trait, 69% of plants with the TTGG genotype were classified as low weight of 50 seeds (A) and 19% as low intermediate (B); and 72% of plants with the CCAA genotype were classified as great weight of 50 seeds (D) and 7% as great intermediate. Of the 57 plants with the lowest weight of 50 seeds (A), one had the CCAA genotype, and none of the 57 plants with the highest weight of 50 seeds (D) had the TTGG genotype.

For SY, plants in F2 with the CC genotype at the SNP EMB-235,706-CT on chromosome 6 tended to be classified as low intermediate (B) to low yield (A), while plants with the TT genotype were classified as high intermediate (C) to high yield (D). For this trait, 51% of plants with the CC genotype were classified as low yield (A) and 36% as low intermediate (B), and 37% of plants with the TT genotype were classified as high yield (D) and 38% as high intermediate (C). Only five of the 57 plants with the lowest yield (A) had the TT genotype, and only two of the 57 plants with the highest yield (D) had the CC genotype.

### Classification in F2:5

F2:5 progenies with different genotypes associated with the SNPs EMB-235,706-CT and EMB-227,730-AG in F2 were also classified based on the phenotypes expressed in F2:5 for the agronomic traits (Table 4). Similar to classification in F2, F2:5 progenies derived from F2 plants with the CC genotype at the SNP EMB-235,706-CT tended to be classified as intermediate (B) to short-cycle, early flowering, and low height (A), and F2:5 progenies derived from F2 plants with the TT genotype as intermediate (C) to long-cycle, late flowering, and high height. F2:5 progenies derived from F2 plants with the same genotype at the SNP EMB-235,706-CT and with the AA genotype at the SNP EMB-227,730-AG tended to have shorter cycles, early flowering, and lower height than plants with the GG genotype. For maturation, 93% of F2:5 progenies with the CCAA genotype in F2 were classified as short-cycle (A) and 7% as short intermediate (B). In comparison, 94% of F2:5 progenies with the TTGG genotype in F2 were classified as long-cycle (D) and 6% as long intermediate (C). None of the 57 shortest-cycle progenies (A) had the TTGG genotype in F2, and none of the 57 longest-cycle progenies (D) had the CCAA genotype in F2.

**Table 4:** Percentage of F2:5 progenies classified according to rank, when considering a specific genotyping of SNPs associated with QTL for soybean agronomic traits evaluated under Asian rust pressure

SNP1^1^	SNP2	Classification^3^	SY^4^	DM	DF	PH	50SW
CC^2^	AA	A	0	93	79	71	0
		B	0	7	14	29	0
		C	29	0	7	0	7
		D	71	0	0	0	93
CC	AG	A	3	74	80	54	0
		B	11	17	14	37	11
		C	32	9	6	9	40
		D	54	0	0	0	49
CC	GG	A	10	14	43	38	33
		B	29	57	47	38	29
		C	33	24	5	19	19
		D	29	5	5	5	19
CT	AA	A	4	49	22	43	0
		B	22	43	61	35	22
		C	35	8	13	13	26
		D	39	0	4	9	52
CT	AG	A	21	8	4	17	19
		B	29	35	38	19	31
		C	31	42	48	46	40
		D	19	15	10	19	10
CT	GG	A	35	0	5	3	46
		B	49	16	19	32	38
		C	11	41	32	30	13
		D	5	43	44	35	3
TT	AA	A	20	0	0	8	7
		B	33	40	13	13	27
		C	33	47	74	52	39
		D	14	13	13	27	27
TT	AG	A	67	0	0	0	48
		B	19	0	0	10	33
		C	14	24	24	33	14
		D	0	76	76	57	5
TT	GG	A	81	0	0	0	81
		B	13	0	0	0	14
		C	6	6	0	0	0
		D	0	94	100	100	0

^1^SNP1: SNP EMB-235,706-CT on chromosome 6 and SNP 2: SNP EMB-227,730-AG on chromosome 16. ^2^Nitrogenous bases: Thymine (T), Cytosine (C), Adenine (A) e Guanine (G). ^3^A: 25% of F2 plants with shorter height, shorter cycle, lower yield and less 50-seed weight, B: low intermediate, C: high intermediate and D: 25% of F2 plants with greater heights, longer cycle, higher yield and greater 50-seed weight. ^4^ SY: seed yield per line, PH: plant height, DM: days to maturity, DF: days to flowering, and 50SW: 50-seed weight

Different phenotypic expressions in F2:5 based on the genotyping of the SNPs EMB-235,706-CT and EMB-227,730-AG in F2 were observed for SY and 50SW. F2:5 progenies with the TT genotype in F2 at the SNP EMB-235,706-CT tended to be classified as low intermediate (B) to low yield (A), while progenies with the CC genotype were classified as high intermediate (C) to high yield (D). F2:5 progenies derived from F2 plants with the same genotype at the SNP EMB-235,706-CT and with the AA genotype in F2 at the SNP EMB-227,730-AG tended to have higher yields than those with the GG genotype. For this trait, 81% of F2:5 progenies derived from F2 plants with the TTGG genotype were classified as low yield (A) and 13% as low intermediate (B). In comparison, 71% of F2:5 progenies derived from F2 plants with the CCAA genotype were classified as high yield (D) and 29% as high intermediate (C). In turn, none of the 57 F2:5 progenies classified as low yield (A) had the CCAA genotype in F2, and none of the 57 F2:5 progenies classified as high yield (D) had the TTGG genotype in F2. Similar results were obtained for 50SW.

The selection of F2 plants for SY with the CC genotype was not suitable, as 24% of the respective F2:5 progenies were classified as intermediate (B) to low-yield (A) (Table 4). On the other hand, 27% of the F2:5 progenies from F2 plants with the TT genotype were classified as intermediate (C) to high yield (D). This inadequate selection, based solely on chromosome 6, was also observed for other traits.

## Discussion

### Disease progression

Disease progression in susceptible and resistant plants in the F2 and F2:5 generations was similar to that reported by Lima et al. (2012). An ASRS above 50% in the fourth evaluation of non-resistant genotypes, along with differences in AUDPC between resistant and non-resistant genotypes, confirmed favorable climatic conditions for disease development in the field. These results also showed the absence of major genes in the population under study. The ASRS and AUDPC values enabled identifying QTL, developing genomic prediction models, and evaluating MAS effectiveness in breeding soybean cultivars under ASR pressure.

### Quantitative trait loci

In QTL mapping, the genes controlling agronomic traits in the F2:5 progenies had d/a ratios near zero, indicating that these genes showed little or no dominance (Table 1). Ribeiro et al. (2008) also did not identify complex genetic interactions, such as epistasis and dominance, regardless of whether *P. pachyrhizi* was present. Given this lack of complex interactions, selection from segregating populations can be an effective strategy for cultivar development.

QTL mapping based on phenotypic values in F2 and F2:5 generations for traits with higher heritability, such as DM, DF, and PH, revealed the presence of consistent QTL on chromosomes 6 and 16 (Table 1). These QTL were also detected for traits with lower heritability, including SY and 50SW, but only through F2:5 phenotypic data.

The three QTL identified between 17,230,927 and 49,111,467 bp on chromosome 6 are located near the E1 gene (Glyma06g23026) situated between 20,006,928 and 20,007,814 bp (Bernard 1971; Watanabe et al. 2004). E1 is a partially dominant gene that controls late flowering and maturity in soybeans under long-day conditions (Bernard 1971). Beyond E1, another gene mapped to chromosome 1 is E7, which is closely linked to the E1 gene (Cober and Voldeng 2001). In working with a soybean RIL population under ASR pressure, Santos et al. (2018) reported that E1 regulated the jt-1 QTL for DF, mapped between 18,534,682 and 27,940,564 bp. A similar influence may apply to the three QTL on chromosome 6 identified in this study. However, the QTL in region 1 of chromosome 6, located closest to E1, was associated with partial dominance for earliness (Table 1).

Several additional genes beyond E1 and E7 controlling DF and DM have been mapped to other chromosomes, including E2 (Bernard 1971), E3 (Buzzell 1971), E4 (Buzzell and Voldeng 1980), E5 (McBlain and Bernard 1987), E6 (Bonato and Vello 1999), E8 (Cober et al. 2010), E9 (Kong et al. 2014), E10 (Samanfar et al. 2017), E11 (Wang et al. 2019)d (Ray et al. 1995). Among these, the E9 locus was mapped to a genomic interval on chromosome 16 delimited by the SSR markers Satt215 (28,589,359 bp) and Satt431 (35,718,538 bp) (Kong et al. 2014). In the present study, a QTL on chromosome 16 associated with agronomic traits was identified between 1,333,772 and 1,402,063 bp (Table 1). Conversely, Santos et al. (2018) did not detect any QTL on this chromosome associated with DF in a RIL population under ASR pressure.

Major genes and QTL controlling flowering often influence agronomic traits beyond flowering and maturity (Wang et al. 2004; Santos et al. 2018; Zhu et al. 2021). QTL on chromosome 6 (Table 1) also showed pleiotropic effects, as observed for the jt-1 QTL (Santos et al. 2018). These authors also identified the jt-2 QTL on chromosome 6, between the markers Sat_251 (bp 39,704,482) and Satt708 (bp 40,461,941), associated with traits directly related to ASR resistance, including the number of uredinia per lesion (NU) and sporulation level (SL). They suggested that jt-2 may be involved in the genetic basis of horizontal resistance to ASR. The results reported by Santos et al. (2018) and those obtained in the present study (Table 1) highlight the importance of the genomic region spanning 17,230,927 to 49,111,467 bp on chromosome 6 in the expression of agronomic traits under Asian soybean rust pressure.

The pleiotropic behavior of QTL on chromosomes 6 and 16 (Table 1) in the present study reflects the observed correlations among traits. Notably, in the absence of ASR, several studies have reported that greater plant height and later maturity are associated with higher yield (Kim et al. 2012; Zhu et al. 2021). In contrast, our findings under ASR pressure revealed that SY in F2:5 was negatively correlated with DM (−0.77), DF (−0.72), and PH (−0.57), and positively correlated with 50SW (0.70). Additionally, DM was negatively correlated with 50SW (−0.75). Supporting these results, Santos et al. (2018) reported similar findings in RIL populations exposed to ASR.

QTL Mapping using F2:5 phenotypic data revealed two QTL on chromosome 6 with R^2^ values of up to 61.0%, and one QTL on chromosome 16 with an R^2^ of up to 31.2%, depending on the trait (Table 1). The R^2^ value of these QTL for SY, DM, DF, and PH was nearly double that of the QTL on chromosome 16. The opposite occurred for 50SW. These differences influenced model performance (Table 2), indicating that chromosome 6 plays a more prominent role in SY, DM, DF, and PH, while chromosome 16 is more important for 50SW.

### Modeling and progeny selection

The use of selected SNPs (i.e. QTL-based markers) in genomic selection (GS) does not represent a fully genome-wide approach, but rather a targeted GS strategy. When the predictive abilities of models from these approaches are comparable, this may suggest that MAS is sufficient for identifying and selecting superior genotypes. Table 2 summarizes the predictive ability of different GS models, including those based on SNPs distributed across all chromosomes as well as models relying on specific subsets of these markers.

Incorporating only SNPs linked to QTL on chromosomes 6 and 16 resulted in genomic prediction models with similar or higher predictive abilities than those obtained using SNPs from all chromosomes (Table 2). For instance, the SNPs EMB-235,706-CT (chromosome 6) and SNP EMB-227,730-AG (chromosome 16) yielded models in F2:5 with higher predictive abilities. In turn, removing SNPs from other chromosomes had no significant impact on prediction ability. Moreover, the inclusion or exclusion of SNPs between the QTL for SY in the F2 population, identified on chromosome 13, for instance, did not alter the predictive ability of the models for this trait (Tables 1 and 2). These results reinforce the role of chromosomes 6 and 16 in regulating key agronomic traits, indicating that MAS may be a suitable approach for selecting superior progenies.

While Santos et al. (2018) detected QTL for DF and other traits only on chromosome 6, selecting F2 plants solely by SNP EMB-235,706-CT led to the unintended loss of F2 and F2:5 genotypes with high yield, early flowering and maturity, short stature, and heavier seeds; instead, suboptimal individuals were selected (Tables 3 and 4). In contrast, including both chromosomes 6 and 16 for MAS across F2 and F2:5 generations improved selection outcomes, as demonstrated by the identification of elite genotypes in F2:5 when MAS was applied at the F2 stage (Tables 1, 2, 3 and 4). This underscores that only relying on chromosome 6 limited genetic gain, whereas combining both chromosomes enhanced agronomic trait selection.

In the F2 generation, SY was positively correlated with DM, DF, and PH, while no correlation was observed with 50SW. F2 plants with the TT genotype at EMB-235,706 exhibited increased plant height and delayed flowering and maturity, which may be undesirable in breeding programs (Tables 3 and 4). Moreover, the low correlation between SY in F2 and F2:5 (−0.24) suggests that MAS based on F2 performance may be ineffective, as phenotypically selected plants in F2 did not lead to the best-performing F2:5 progenies. In contrast, SY in F2:5 was negatively correlated with DM, DF, and PH, and positively with 50SW. Plants with the CC genotype at EMB-235,706-CT (chromosome 6) and AA at EMB-227,730-AG (chromosome 16) had higher SY and 50SW, and lower DM, DF, and PH, consistent with observed trait correlations (Table 4). The opposite was observed in genotypes with the TTGG combination. This differentiation was notably achievable when major-effect genomic regions were present, despite some QTL showing only moderate R² values (< 30%) (Tables 1 and 3, and 4).

Carvalho et al. (2025) conducted genomic selection by developing prediction models based on genotypic data from F2 plants and phenotypic evaluations of F2:5 progenies. In the present study, we demonstrated that only SNPs associated with QTL significantly influenced the predictive ability of these models, thereby simplifying the modeling framework and supporting the potential application of MAS. Furthermore, MAS applied in the F2 generation proved effective in selecting superior F2:5 progenies, particularly when major-effect genomic regions were present, despite some QTL exhibiting only moderate R² values. Information regarding the inheritance patterns of the evaluated traits is also presented.

The data presented in Tables 1, 2, 3 and 4 were generated from evaluating 230 F2 plants derived from a single biparental population grown in a single environment (Londrina, PR, Brazil). Furthermore, genomic prediction was conducted using repeated k-fold cross-validation within the same population, a procedure which may overestimate predictive ability (Table 2). Under these conditions, predictive ability reflects within-population accuracy rather than true-forward predictive performance. Nevertheless, the use of a single biparental population enabled identifying SNPs and implementing MAS at the F2 stage, facilitating the selection of superior F2:5 progenies under ASR pressure (Table 4). The comparable predictive performance observed between genome-wide GS and GS based on selected SNPs (QTL-based) underscores the relevance of SNP markers for selection, irrespective of their individual effect sizes. Additionally, higher-yielding plants tended to be earlier maturing, shorter in stature, and exhibited greater 50-seed weight. In this context, yield is associated with less complex traits and is consequently less influenced by environmental variation. This likely accounts for the relatively small number of SNPs detected and the effectiveness of MAS in identifying superior F2:5 progenies.

In this study, genotypic data from the F2 population and phenotypic data from the F2:5 population demonstrated the effectiveness of MAS in identifying superior F2:5 soybean progenies under ASR pressure. Genomic selection models incorporating SNPs linked to QTL on chromosomes 6 and 16 performed comparably to, or better than, genome-wide GS models. QTL on chromosome 6 were more strongly associated with seed yield, days to maturity, days to flowering, and plant height, whereas chromosome 16 was primarily associated with 50-seed weight. The use of targeted SNPs enabled selecting progenies with enhanced yield, earlier maturity, reduced plant height, and increased 50-seed weight, particularly when major genomic regions with strong effects were present, even though some QTL accounted for less than 30% of the phenotypic variation. These findings support the use of MAS as a complementary approach for developing soybean cultivars with improved agronomic performance under ASR pressure.

## Data Availability

The datasets generated during and/or analyzed during the current study are available from the corresponding author upon reasonable request.
